# Insights into Antibody-Mediated Alphavirus Immunity and Vaccine Development Landscape

**DOI:** 10.3390/microorganisms9050899

**Published:** 2021-04-22

**Authors:** Anthony Torres-Ruesta, Rhonda Sin-Ling Chee, Lisa F.P. Ng

**Affiliations:** 1 A*STAR Infectious Diseases Labs (A*STAR ID Labs), Agency for Science, Technology and Research (A*STAR), Singapore 138648, Singapore; anthony_torres@IDLabs.a-star.edu.sg (A.T.-R.); rhonda_chee@IDLabs.a-star.edu.sg (R.S.-L.C.); 2 Department of Biochemistry, Yong Loo Lin School of Medicine, National University of Singapore, Singapore 117596, Singapore; 3 Institute of Infection, Veterinary and Ecological Sciences, University of Liverpool, Liverpool L69 3BX, UK

**Keywords:** alphavirus, antibody, immunity, alphavirus vaccine

## Abstract

Alphaviruses are mosquito-borne pathogens distributed worldwide in tropical and temperate areas causing a wide range of symptoms ranging from inflammatory arthritis-like manifestations to the induction of encephalitis in humans. Historically, large outbreaks in susceptible populations have been recorded followed by the development of protective long-lasting antibody responses suggesting a potential advantageous role for a vaccine. Although the current understanding of alphavirus antibody-mediated immunity has been mainly gathered in natural and experimental settings of chikungunya virus (CHIKV) infection, little is known about the humoral responses triggered by other emerging alphaviruses. This knowledge is needed to improve serology-based diagnostic tests and the development of highly effective cross-protective vaccines. Here, we review the role of antibody-mediated immunity upon arthritogenic and neurotropic alphavirus infections, and the current research efforts for the development of vaccines as a tool to control future alphavirus outbreaks.

## 1. Introduction

Mosquito-borne alphaviruses are Group IV viruses that belong to the family Togaviridae [1]. They are enveloped, positive-sense, single-stranded RNA viruses with a size of ≈70 nm bearing a ≈11.7 kilobases genome which encodes four non-structural proteins (nsP1, nsP2, nsP3 and nsP4) that serve as the virus’ replication machinery, and five structural proteins (capsid, E3, E2, 6K and E1) that participate in the envelope assembly process [1]. Clinically, alphavirus infections in humans results in the development of viremia followed by an onset of febrile symptoms [2]. The development of inflammatory conditions compromising joints and muscle tissues has been associated to arthritogenic alphaviruses such as chikungunya virus (CHIKV), O’nyong nyong virus (ONNV), Mayaro virus (MAYV), Ross River virus (RRV), Semliki Forest virus (SFV) and Sindbis virus (SINV) with records of persistent polyarthralgia in a fraction of patients. Conversely, neurotropic alphaviruses such as Eastern Equine Encephalitis virus (EEEV), Western Equine Encephalitis virus (WEEV) and Venezuelan Equine Encephalitis virus (EEEV) have been linked to the induction of lethal encephalitis in humans and animals [3,4].

Historically, alphaviruses have a proven record of causing massive outbreaks in susceptible populations [5,6,7,8]. Additionally, the appearance of mutations favoring their ecological fit to new vectors has fueled alphavirus propagation worldwide [9,10]. A clear example of their potential as a health threat is the re-emergence of CHIKV in 2004 after a hiatus of more than 50 years since its discovery [5]. More recently, tropical emerging alphaviruses such as ONNV and MAYV are believed to have the potential to become future major epidemics [11,12,13]. This is due, in part, to the lack of robust diagnostic tests to differentiate alphavirus infections from other febrile tropical diseases and the absence of continuous epidemiological surveillance masking their real potential for spread beyond endemic areas [14,15,16].

Although alphavirus infections are generally not life threating the economic and social costs incurred during outbreaks are thought to be high [17,18,19]. Moreover, the lack of approved treatments leaves management of alphavirus infections to supportive care [20]. Interestingly, a body of work suggests that the alphavirus infection triggers potent humoral responses in exposed populations which seem to confer protection against re-infection [21]. Therefore, a better understanding of the antibody responses against alphaviruses is crucial for the development of vaccines, which would represent a big advantage in the control of alphavirus infections.

## 2. Antibody-Mediated Alphavirus Immunity

### 2.1. Virus-Specific Antibody Kinetics Upon Natural Infection with Alphaviruses

The current knowledge on the role of antibody-mediated immunity upon viral infection has been gathered from cohort studies following major alphavirus outbreaks. Serological surveys following CHIKV re-emergence in 2004 reported the quick development of IgM responses between five to seven days post-illness onset (PIO) [22,23]. IgM is generally detectable for up to three months post-infection [24,25,26]. However, long-lasting IgM has been often reported in patients with long-term CHIKV-induced polyarthralgia, which might indicate a constant antigenic stimulation due to viral persistence [27]. After the initial detection of IgM antibodies, IgG seroconversion reportedly occurs between 4 to 10 days PIO taking over as the main immunoglobulin detected in serum [22,28]. Notably, IgG3 antibodies become the dominant IgG subtype produced upon infection and have been associated to efficient viral clearance and protection against chronic CHIKV symptoms [23]. Importantly, IgG responses persist for several years and might be potentially lifelong [29].

ONNV and MAYV, both closely-related to CHIKV, are re-emerging arthritogenic alphaviruses believed to be confined to sub-Saharan Africa, and Latin America, respectively [6,11,12,15]. Following the largest ONNV outbreak in Uganda involving more than two million cases between 1959–1962 [6,30], the induction of potent neutralizing antibodies was described [31]. The first study cohort that evaluated IgM kinetics upon ONNV infection in Uganda [32] reported the appearance of IgM antibodies during the second week PIO which remained elevated for two months. In contrast, reports from imported ONNV cases in Europe described detectable IgM levels as early as five days PIO [33,34]. ONNV-specific IgG levels are increased in serum after the third week and remain high beyond two months PIO [11,34]. However, whether IgG responses are long-lasting remains unknown. Similarly, endemic MAYV infections are characterized by the early appearance of IgM antibodies (3–8 days PIO) that might last for one to three months [35,36]. IgG becomes detectable around 4–10 days PIO [35] and remains elevated after 6–12 months [37,38]. Interestingly, unlike ONNV and CHIKV infections, persistent arthralgia has been reported in more than half of MAYV-infected individuals and although MAYV-specific antibody responses are critical for disease resolution it is seemingly insufficient to protect patients from the development of chronic joint manifestations [39].

Other alphaviruses linked to continuous small outbreaks associated with arthritic manifestations in human populations are RRV and SINV. RRV is endemic to Australia and is responsible for approximately 4000–5000 cases annually [40]. Typically, antibody kinetics upon RRV infection are characterized by the development of IgM titers between 7–10 days PIO, peaking at two to three weeks and lasting for 1–3 months [41,42]. IgM response rapidly declines after three weeks PIO as IgG becomes dominant [42,43]. Interestingly, IgM persistence has been reported in some RRV cohorts [41]. In one study [44], 19/116 (16.4%) of participants had detectable IgM levels that lasted between seven months to eight years PIO. Likewise, less prevalent SINV has also been linked to the development of persistent virus-specific IgM levels. Although, generally, the antibody response upon SINV infection generates IgM antibodies after 6–9 days PIO and IgG antibodies after 9–14 days, some reports described the presence of detectable IgM levels up to four years suggesting active viral replication [45,46,47]. The clinical relevance of persistent IgM levels following RRV and SINV infection is yet to be determined.

Neurotropic alphaviruses such as EEEV, WEEV and VEEV cause sporadic cases of human encephalitis in the Americas [4]. While the natural reservoirs for these viruses are primarily birds and equines, humans are susceptible to infection when the enzootic cycle of transmission leaks into mosquito populations with a wide range of hosts [48]. Given that human cases are rare, there is a lack of information regarding the development of antibody responses upon natural infections by neurotropic alphaviruses. In a paired serology study [49], virus-specific antibody responses were profiled in a cohort of 20 EEEV and 17 WEEV-infected patients. IgM antibodies were observed as early as 1 PIO, peaking after 1–2 weeks and remaining detectable for up to three months. In contrast, IgG responses appeared during the second week PIO and remained elevated until the end of the follow-up period.

### 2.2. Experimental Evidence of the Role of Antibodies in Alphavirus Immunity

To better understand the role of antibody-mediated immunity upon alphavirus infection, several animal models have been used allowing the detailed examination of the cellular compartments responsible for the initiation of humoral immunity. The role of B cells in alphavirus immunity has been described in experimental CHIKV infections. Inoculation of µMT mice (lacking mature B cells) with CHIKV resulted in higher viremia that persisted up to 402 days post-infection (DPI). In contrast, infected wild type (WT) mice were able to control the virus during the second week post-inoculation [50]. Similar findings were reported in other studies, where mouse strains lacking B cells (µMT, Rag1, Rag2/IL2rg, NRG) infected with CHIKV displayed increased and persistent viremia for up to 515 DPI [51,52].

B cells also play an important role in alphavirus-induced encephalitis. Although SINV infections in humans are known to cause arthritic manifestations, SINV has been frequently used as a model of alphavirus-induced encephalomyelitis in adult immunocompetent mice given the virus ability to infect neurons [53]. Intracerebral inoculation of SINV in µMT and severe combined immunodeficiency (SCID) mice resulted in defective viral clearance from the brain, brain stem and lumbar spinal cord, virus persistence and recrudescence compared to WT mice [54]. The individual contributions of IgM and IgG antibodies to SINV clearance from brain tissues were assessed in another study [55] where infection in AID^−/−^ (unable to produce IgG), sIgM^−/−^ (unable to produce IgM) and AID^−/−^ sIgM^−/−^ double-knockout mice resulted only in AID^−/−^ sIgM^−/−^ being unable to control infection efficiently suggesting that either IgM or IgG antibodies are sufficient to clear SINV from the central nervous system (CNS). Similar results were obtained in SFV models of encephalitis where infection of µMT [56], SCID [57] and nude mice with impaired antibody switching [58] led to viral persistence.

Infiltrating virus-specific B cells were observed in infected tissues in a murine model of SINV-induced encephalitis [59,60]. Following intracranial virus inoculation, expansion of IgM-secreting plasmablasts was reported in the cervical lymph nodes. Infiltration of CD19+ B cells occurred between 3–7 DPI and coincided with the starting of viral clearance. During the clearance of persistent viral RNA (from 8–80 DPI), the accumulation of SINV-specific IgG and IgA-secreting B cells was observed being associated with increased SINV antibody titers over time [60]. In a subsequent study, it was reported that the brain microenvironment during the early stages of SINV infection facilitates the migration, differentiation, expansion and long term survival of SINV-specific B cells [59].

Follicular helper T cells (T_FH_) are a subset of CD4 T cells involved in the activation of B lymphocytes and the establishment of robust antibody responses following antigen stimulation. T_FH_ promotes B cell differentiation, isotype switching and affinity maturation. In experimental CHIKV infections, the use of CD4-deficient mice ruled out the role of CD4 T cells in viral clearance from infected tissues [61]. However, one study demonstrated impaired IgM and IgG (IgG2c, IgG1, and IgG2b) production in mice lacking CD4 T cells following CHIKV inoculation [62]. Albeit reduced virus-specific antibody levels, the neutralizing capacity of sera from virus-infected CD4-deficient mice was marginally affected [62]. Likewise, another study showed similar results upon CHIKV inoculation of MHCII^Δ/Δ^ mice (defective of T_FH_) [51]. MHCII^Δ/Δ^ animals were unable to generate IgG1 antibodies and produced ≈100 fold lower IgG2c levels than WT controls. Nonetheless, MHCII^Δ/Δ^ mice were still able to control virus infection [51]. The generation of virus-specific neutralizing antibodies in MHCII^Δ/Δ^ mice suggests a T-cell independent B cell activation characterized by the inability to generate memory B cells. Whether CHIKV-specific antibody responses in mice lacking CD4 T cells are long-lasting remains to be elucidated.

### 2.3. Viral Antigenic Regions Targeted by Neutralizing Antibodies

The notion of targeting humoral immunity as a therapy against alphavirus infection has been investigated since the late 1930s following the isolation of EEEV, WEEV and VEEV. In a series of seminal studies involving immunization of guinea pigs [63,64,65,66], the subcutaneous inoculation of live EEEV and WEEV strains protected guinea pigs from lethal intracranial infection [63]. Additionally, it was observed that immunization with formalin-inactivated virus strains induced the production of neutralizing antibodies at a comparable level than animals immunized with live viruses [64,65,66]. Subsequent studies reported that passive transfer of hyperimmune rabbit serum protected mice, guinea pigs and rabbits from WEEV infection [66,67]. Similarly, passive serum transfer was shown to be effective at protecting mice from the development of neurological complications upon infection with a neuroadapted strain of SINV [68,69]. Comparable observations were reported in experimental infection models of VEEV [70], CHIKV [71,72], RRV [73] and SFV [74].

**Figure 1:**
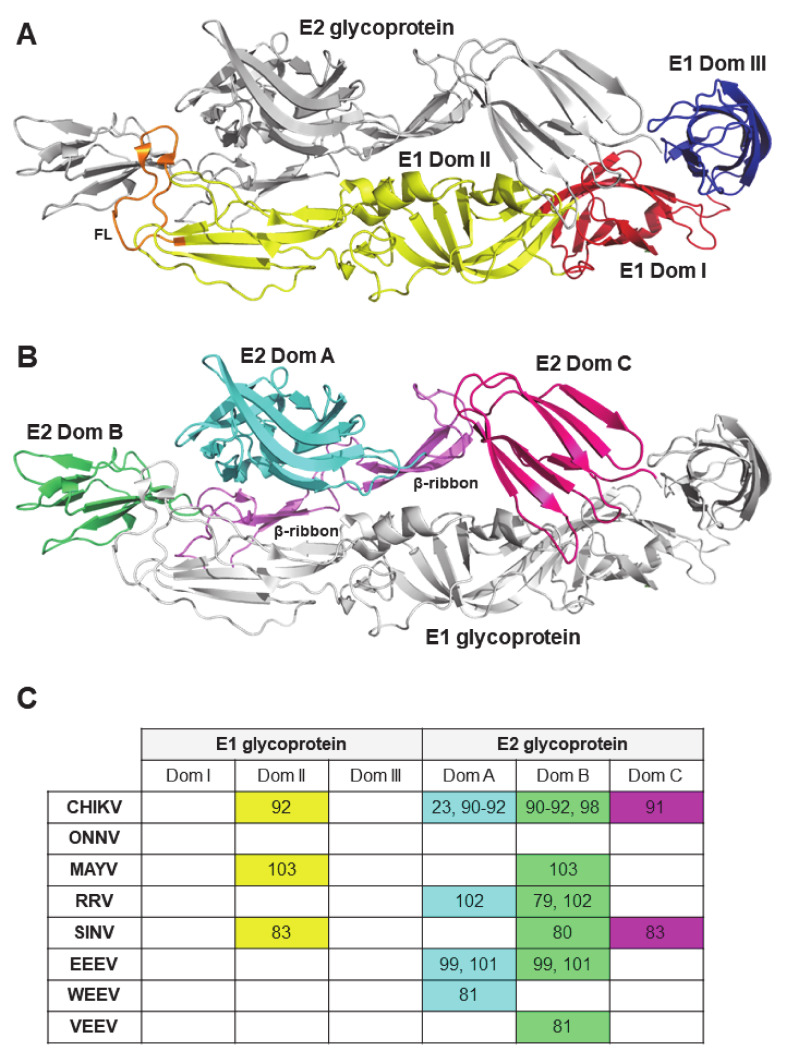
Structure of the alphavirus E1-E2 heterodimer. Ribbon diagram (PDB: 3N41) highlighting (**A**) E1 glycoprotein (domain I: red, domain II: yellow, domain III: blue, fusion loop FL: orange, E2: grey) and (**B**) E2 glycoprotein (domain A: cyan, domain B: green, domain C: pink, beta-ribbons: purple, E1: grey). (**C**) Table summarizing reported antibody binding regions in the E1 and E2 glycoproteins of arthrogenic and neurotropic alphaviruses. Numbers in the table refer to in-text citations describing such binding sites (See Reference list). Background color matches protein doimains depicted in (**A**) and (**B**). To assess for the degree of conservation among common antigenic regions across alphaviruses a sequence aligment analysis was conducted (See Supplementary Figure S1).

The first attempts in identifying the exact structural regions, recognized by most neutralizing antibodies produced upon infection, were conducted in experimental infection models of alphavirus encephalitis. Structurally, the envelope of an alphavirus virion has a T = 4 icosahedral symmetry [75]. E1 and E2 are two envelop surface glycoproteins exposed in the viral spike as a heterodimer [75] (Figure 1). It is believed that the E1-E2 heterodimer interacts with host receptors thus mediating viral entry [75]. Additionally, the E1 and E2 glycoproteins were postulated as highly immunogenic regions since their location in the spike facilitates antigenic recognition. In line with this, early works mapped antigenic sites involved in VEEV, SINV and SFV neutralization to the E1 and E2 proteins using competitive binding assays but the exact amino acid sequences were not determined [76,77,78]. Later, a major antigenic region involving three epitopes important in the neutralization of RRV was identified in the E2 protein (incorporating residues 216, 232 and 234) [79]. Similarly, analysis of antibody escape variants determined important antigenic regions between amino acids 181 and 216 on the E2 protein of SINV [80]. A major neutralization domain was also identified between residues 182–207 for VEEV [81].

Following CHIKV reemergence in 2004 several reports identified major linear antigenic sites in the CHIKV E2 protein that induced the production of potent neutralizing antibodies. Using a CHIKV proteome-wide screening approach, a single linear peptide located at the N-terminus of the E2 glycoprotein, E2EP3, was reported as strongly recognized by convalescent CHIKV patients from different cohorts [23]. Furthermore, experimental CHIKV infection in mice and non-human primates (NHP) validated E2EP3 as an immunodominant linear epitope inducing potent neutralizing antibodies [23,62,82]. Interestingly, mice immunization with E2EP3 alone reduced joint swelling and viremia upon CHIKV challenge [23]. In another study focusing on human antibody responses to SINV in cohort from Finland, 6 linear epitopes, located in the capsid, E2, E1 and PE2 (uncleaved E3-E2) proteins, were reported [83]. Three of these epitopes were located to the glycoprotein spike complex between the residues 209–226 of E1 (E1-P5), 273–290 (E2-P3) and 308–325 (E2-P4) of E2 [83]. Interestingly, the E2EP3 equivalent of SINV remained non-reactive suggesting that antibody kinetics against linear E2EP3 between populations exposed to CHIKV and SINV might differ [83].

The development of mouse and human monoclonal antibodies against different alphaviruses helped further the understanding of antigenic responses upon infection by the identification of conformational epitopes. Early works have shown the therapeutic value of mouse monoclonal antibodies in models of alphavirus encephalitis by SINV [84,85,86,87,88], SFV [56,57,89] and VEEV [78]. Interestingly, it was observed that neutralizing monoclonal antibodies target antigenic regions in the E2 protein. Whereas, non-neutralizing antibodies bind to the E1 protein, yet both are able to confer protection upon alphavirus infection, thereby suggesting other mechanisms of protection in vivo besides virus neutralization [48]. Several monoclonal antibodies targeting both E1 and E2 proteins have been reported in the context of arthritogenic alphavirus infection. Mouse monoclonal antibodies targeting the A and B domain of E2 and the domain II of E1 [90,91,92] and the capsid protein [93,94] have been reported for CHIKV. Likewise, human anti-CHIKV monoclonal antibodies were found to target conformation epitopes in the E2 glycoprotein A (containing a putative RBD [95]) and B (shielding the fusion loop in E1 [96]) domains and proved therapeutic value in experimental NHP infections [90,97,98]. Monoclonal antibodies recognizing epitopes predominantly between residues 58–80 (domains A) or residues 180–215 (domain B) of the E2 glycoprotein have been also reported in the context of SINV [83], VEEV [81], EEEV [99,100,101], RRV [102] and MAYV [103].

The combined evidence suggested the existence of common antigenic sites in the viral spike across alphaviruses, particularly in the E2 protein. These sites are likely required for interaction with host cell receptors suggesting that antibody binding might inhibit infection during viral attachment, entry, fusion or egress [90]. In line with this, a recent study reported the discovery of Mxra8, a cell adhesion molecule, as a host receptor required for viral entry of multiple arthritogenic alphaviruses [104]. Genetically altering mouse or human Mxra8 resulted in diminished infection, conversely, overexpression of Mxra8 in cell lines increased infection rates by CHIKV, ONNV, MAYV and RRV [104,105]. Interestingly, mutagenesis experiments suggested E2 domains A and B as the putative binding site for Mxra8 [104]. This notion was later confirmed by cryo-electron microscopy images of Mxra8 bound to CHIKV [106,107]. Mxra8 sits onto a cleft formed by two contiguous CHIKV E2-E1 heterodimers in one trimeric spike while engaging a neighboring spike [106]. It is believed that this interaction works against the virus by obstructing viral fusion [106]. Importantly, human neutralizing antibodies that recognize regions of the A domain of E2 inhibited the binding of Mxra8 supporting the interactions determined in the cryo-EM atomic model. Notably, Mxra8 seems to not be a receptor for neurotropic alphaviruses [104]. The alignment of CHIKV residues involved in Mxra8 binding reveled a degree of conservation in arthritogenic alphaviruses (44%), but diverged from neurotropic Alphaviruses (14%) which might explain the negative results in the context of SINV, EEEV, WEEV and VEEV infections [106]. In summary, the characterization of alphavirus antigenic epitopes has proven beneficial to pave the way for the development of antibody therapies and vaccines.

## 3. Alphavirus Vaccine Development

**Figure 2:**
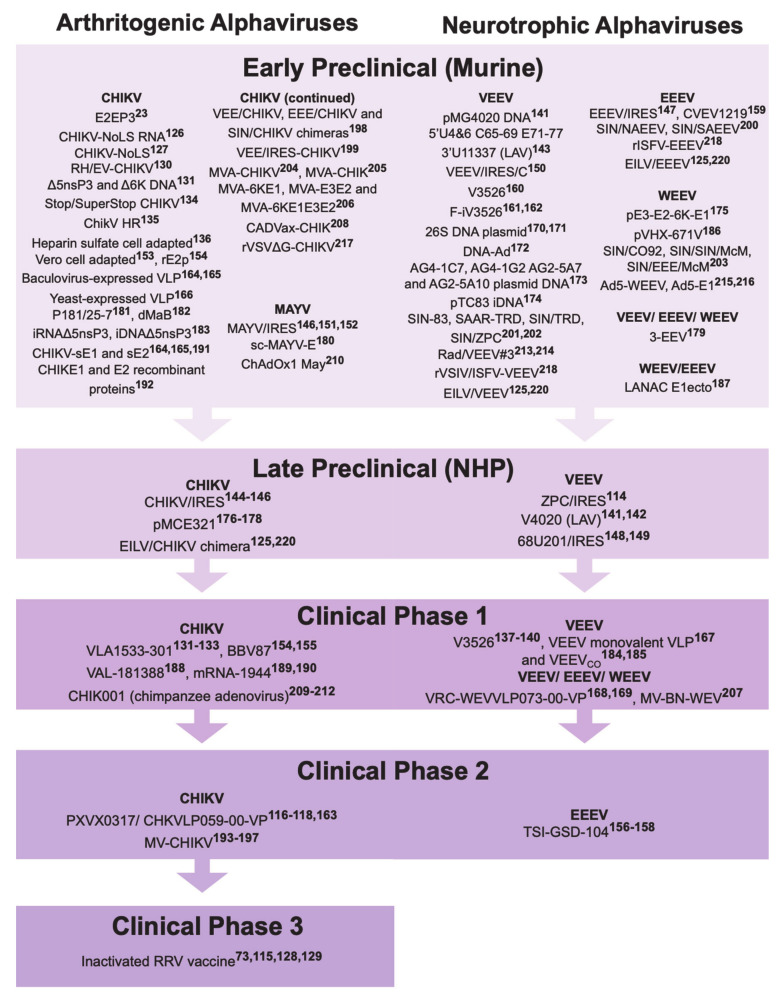
An outline of the current vaccine options against arthritogenic (left panel) and neurotropic (right panel) alphaviruses. Most of these vaccine candidates are currently under preclinical testing (early preclinical—vaccine candidates tested in mouse models; late preclinical—vaccine candidates currently under testing in non-human primates (NHP)), while a minority of them are currently undergoing clinical trials (Phase 1, 2 or 3). LAV; live-attenuated virus; VLP, virus-like particle; SIN, Sindbis virus; ISFV, Isfahan virus; May, Mayaro virus; EILV, Eilat virus, VSV/VSIV, vesicular stomatitis virus; MV, measles virus; MVA, modified vaccinia virus Ankara. Data curated from literature reported through February 2021. Numbers in superscript refer to reference numbers (See Reference list [23,73,114,115,116,117,118,119,120,121,122,123,124,125,126,127,128,129,130,131,132,133,134,135,136,137,138,139,140,141,142,143,144,145,146,147,148,149,150,151,152,153,154,155,156,157,158,159,160,161,162,163,164,165,166,167,168,169,170,171,172,173,174,175,176,177,178,179,180,181,182,183,184,185,186,187,188,189,190,191,192,193,194,195,196,197,198,199,200,201,202,203,204,205,206,207,208,209,210,211,212,213,214,215,216,217,218,219,220]).

Recent decades have seen increased rates of geographic dispersal of arboviral re-emergence, due to factors such as growth of global transportation, urbanization and failure of mosquito control [108,109,110,111]. Given that humans appear to be the only amplification hosts and viral reservoir during urban transmission [112,113], another effective means of controlling the spread of infection is through vaccination. While there are currently no licensed or approved vaccines available for alphaviruses, a multitude of approaches have been used to develop vaccine candidates capable of, not only generating high levels of antibodies, but also providing long-lasting protection, with the ease of administration and production requirements. Multiple methods such as live-attenuated viruses, inactivated viruses, virus-like particles (VLP), recombinant subunit vaccines and chimeric vaccines have been explored for vaccine options (Figure 2 and Table 1).

### 3.1. Live-Attenuated Vaccines

With the development of alphaviruses in reverse genetic systems, more research has been focused on the rational design of live-attenuated vaccines [221,222] in overcoming potential issues, such as genetic reversion mutations in vaccines [223,224], with highly specific mutations or alterations of the original parental virus genome. In addition, not only are the safety profiles of these vaccines is greatly improved, protection with a only single dose is also achieved [225].

An engineered live-attenuated option for alphavirus vaccine design involves the rational design of downregulating the expression of particular structural proteins with the introduction of a picornavirus (encephalomyocarditis virus) internal ribosome entry site (IRES) into the viral genome. For example, this is demonstrated in a VEEV vaccine candidate, ZPC/IRES, where the expression of the capsid protein is minimalized by translocating its gene to a separate opening reading frame downstream of the envelope glycoprotein genes and interrupting its expression with the introduction of a IRES [114,226]. However, the highly immunogenic envelope glycoproteins E3-E1 were not manipulated, but the insertion of IRES into the genome would functionally alter the host range as replication of the live virus is restricted in mosquitoes.

ZPC/IRES is based on a full-length clone of a wild type VEEV subtype ID from Zulia state, Venezuela from a sentinel hamster exposed in a tropical lowland. CD-1 mice immunized with 10^5^ PFU of ZPC/IRES developed strongly neutralizing antibodies, with PRNT80 of average reciprocal titer of 324 by 20 weeks post immunization. Subsequently, when immunized mice were challenged with the lethal VEEV subtype IC strain 3908 (10^5^ PFU, subcutaneous or 10^4^ PFU, aerosol route) 4 weeks after immunization, all mice retained their weight and failed to show any signs of disease and survived, compared to mock-vaccinated mice which succumbed to the lethal infection. Additionally, the study tested the vaccine in a NHP immunization-challenge model in the same study. Vaccinated NHPs had PRNT80 values of 160 to 320, and all vaccinated NHPs were protected against viremia upon challenge with VEEV 3908 strain. Using the VEEV ID strain ZPC738 as the vaccine backbone, which is closely related to subtypes IAB and IC, the authors had aimed to develop an IRES-based, live-attenuated vaccine candidate that could possibly protect against other subtypes of VEEV, given that previous attempts to create a vaccine candidate based on the VEEV subtype IAB V3526 vaccine could not significantly protect against aerosol challenge with a subtype IE VEEV strain [226]. Nonetheless, this hypothesis was not pursued in the study, and it would have been curious to learn whether the ZPC/IRES-immunized animals would be protected from a lethal challenge with VEEV of other subtypes, such as subtypes IA/B and IE.

### 3.2. Inactivated Vaccines

The inactivated Ross River virus (RRV) vaccine is the most developed and advanced vaccine candidate, having been rigorously tested in both preclinical and up to Phase 3 clinical trials. The Vero cell culture-derived whole-virus RRV vaccine was first produced from a viral seed derived from an RRV isolate from a serologically confirmed case of RRV disease in Queensland, Australia, and subsequently inactivated by sequential formalin and UV light treatment after harvest [227]. In pre-clinical testing of the RRV vaccine, CD-1 mice were given two doses of the inactivated RRV vaccines at different experimental doses 28 days apart, without the use of an adjuvant in its formulation. Upon challenge with 10^6^ TCID50 of the mouse-virulent RRV prototype strain T48 at 42 days post immunization, a vaccine dose beyond 0.625 μg provided almost complete protection against viremia development at 1-day post challenge. Interestingly, the possible antibody-dependent enhancement by RRV vaccination by a closely related alphavirus infection was investigated, where viremia in CHIKV LR2006 OPY-1-infected-RRV vaccinated mice was significantly reduced as compared to the control. In this heterologous situation, partial cross protection was observed, but the presence of sub-protective levels of RRV vaccine-induced antibodies prevented the enhancement of CHIKV replication [73].

Subsequently, a randomized Phase 3 clinical trial for the RRV vaccine was conducted in Australia to investigate the safety and immunogenicity of the vaccine in a large cohort of 1755 healthy younger adults aged 16 to 59 years and 209 healthy older adults aged > 60 years [115]. The 2.5 μg Al(OH)3-adjuvanted vaccine was given over three doses (subsequent boosts at 3 weeks and 6 months). The majority of participants in the younger and older adult populations had seroprotective uNT titers after three immunizations with the whole-virus RRV vaccine, and titers of serum IgG antibodies after three immunizations were higher than the serological IgG ELISA titer threshold associated with protection after natural infection with RRV [115]. While the RRV vaccine had been brought forward to Phase 3 clinical trials, and despite the vaccine demonstrating safety and efficacy, it was not considered financially viable to manufacture, despite Queensland recording its largest and worst epidemic between 2014 to 2015 [228,229]. In addition, given that the cost of vaccine trials is hard to justify for a disease that occurs only in Australia and Papua New Guinea, and where the disease is never fatal, efforts to further develop the RRV vaccine were unfortunately halted.

### 3.3. Virus-Like Particles (VLPs)

The VRC-CHKVLP059-00-VP is one of the first potential new CHIKV vaccines to reach advanced development with human clinical testing [116,117]. The CHIKV envelope gene cassette encoding the native polypeptide, E3-E2-6K-E1, of CHIKV strains 37,997 (West African genotype) and LR2006 OPY-1 were inserted into a cytomegalovirus CMV/R expression vector and subsequently transfected into 293T human kidney cells [118]. The resulting VLP product is a CHIKV VLP that is structurally identical to its infectious counterpart (given that structural genes are intact), but is not infectious as its genetic material is removed. While the CHIKV 37997 strain yielded approximately 100 times more VLPs than that from strain LR2006 OPY-1, the former strain was subsequently used to produce the VLPs. Nonetheless, given that the ECSA lineage was responsible for the ongoing outbreak at the time of development, the high degree of amino acid similarity between the two CHIKV strains suggested that the vaccine would be protective against viruses of other genotypes. However, it would have been curious to characterize a VLP produced from a CHIKV strain of the ECSA lineage, given that it is the strain responsible for recent Chikungunya epidemics all around the world [119,120,121,122,123].

BALB/c mice immunized with two doses of 19 μg of CHIKV VLPs intramuscularly generated the highest neutralizing titer against both the homologous strain 37,997 and the heterologous strain LR2006 OPY-1. In addition, NHPs immunized with 20 μg of VLPs developed substantial neutralizing activity to both homologous and heterologous strains after primary immunization. Interestingly, even though the VLP was made from CHIKV 27,997 strain, there was slightly better neutralization of LR2006 OPY-1 compared to 37,997 in both mice and NHPs. The study speculated that this is suggestive that the LR2006 OPY-1 virus may present a conserved epitope to the immune system better than the 37,997 virus. When total IgG antibodies were passively transferred from immunized NHPs to defective type 1 interferon signaling immunodeficient mice (Ifnar1^−/−^), these recipient mice did not develop detectable viremia and all survived a lethal challenge with CHIKV LR2006 OPY-1. This indicated that the humoral immune responses induced by the CHIKV VLPs confer protection against CHIKV infection [124].

This promising data eventually led to further testing in clinical trials—phase 2 studies were concluded and reported in 2020 [117]. The randomized phase 2 clinical trial included 400 healthy adults in outpatient clinics in 6 countries in the Caribbean. Two doses of 20 μg of CHIKV VLP, termed as VRC-CHKVLP059-00-VP in clinical trials, were administered 28 days apart via intramuscular injection. Vaccine-induced humoral immune responses in individuals were comparable with titers from participants vaccinated in the phase 1 trial [116], and serum collected from participants in the phase 1 trial induced neutralizing antibodies against all 3 genotypes of CHIKV. Interestingly, while the phase 2 trial aimed to only enroll CHIKV seronegative participants, 20% of the cohort (in particular, participants from 2 study sites—Dominican Republic and Haiti) were retrospectively found to be seropositive at baseline on the day of the study enrolment, possibly due to seroconversion between screening and study enrolment. A post-hoc analysis demonstrated that the VLP was immunogenic among these seropositive recipients, but a significant difference was observed between the geometric mean ratio between seropositive and seronegative vaccine recipients. Further studies on this specific group of participants to understand the possible effects of seropositivity and efficacy or protection of the CHIKV VLP administered will be interesting, rendering the need for additional clinical trials [117].

### 3.4. Chimeric Viruses

Another option in vaccine development in providing high levels of immunity is the use of a virus-vector system that utilizes an avirulent backbone, but incorporates the expression of viral genetic elements, such as the chimeric vector system for producing foreign gene products.

The insect-only host-restricted Eilat virus (EILV) has recently also been utilized as a chimeric backbone to replace the structural open reading frame with that of EEEV, VEEV or CHIKV [125]. Given that EILV is unable to replicate in vertebrate cells and in brain tissues of infant mice, this enhances the safety aspect of the vaccine, and thus, also serves as a inactivated vaccine, which enhances the expression of particular immunogenic proteins. Separately, the monovalent EILV/EEEV and EILV/VEEV vaccines were efficacious in their protection against lethal alphavirus challenge—immunized CD-1 mice had a high seroconversion rate observed post vaccination and were highly protected from lethal EEEV-FL93 or VEEV-3908 challenge. Compared to mock-vaccinated animals, EILV/EEEV or EILV/VEEV immunized animals had little or no weight loss and were protected from disease. More importantly, a trivalent vaccine containing the EILV/EEEV, EILV/VEEV and EILV/CHIKV chimeras was formulated and assessed if the vaccine could provide protection against lethal challenge with multiple alphaviruses. A single trivalent dose of EILV/VEEV, EILV/EEEV, and EILV/CHIKV elicited neutralizing antibodies against all three viruses and provided >80% protection against VEEV and EEEV lethal challenge. Collectively, this work showed safety combined with strong immunogenicity and ease of production, making the use of the EILV alphavirus chimeric vaccine platform promising and attractive [125]. The use of a trivalent vaccine candidate also serves as a proof-of-concept to show practicality and increases its potential as a vaccine against neurotropic alphaviruses.

### 3.5. Nucleic Acid-Based Vaccines

Commonly known as the ‘third-generation vaccine’, RNA and DNA vaccines form one of the latest vaccine approaches for alphaviruses. The risk of infection from receiving a vaccine is minimal, given the safety associated with the nucleic acid product [230]. In addition, as some vaccines have been shown to have poor immunogenicity due to the lack of uptake or the need for adjuvants [231], much research over the past decades have explored the design of constructs and novel delivery technologies to overcome these issues. In order to overcome several issues related to traditional vaccine development, such as high cost and difficulty in production, RNA has emerged as an effective platform to deliver vaccines using nanoparticle delivery vehicles, such as liposomes [232,233,234].

A RNA vaccine against CHIKV involves the delivery of the self-replicating RNA genome of the live attenuated CHIKV-NoLS virus with CAF01 liposomes [126]. The mutation in the nucleolar localization sequence (NoLS) in the capsid protein of CHIK-NoLS was previously shown to significantly attenuate viral replication [127]. In the same study, C57/BL6 mice immunized with one dose of CHIKV-NoLS were fully protected from CHIKV infection [127]. In immunodeficient AG129 mice, a single dose of CHIKV-NoLS RNA delivered with CAF01 generated CHIKV-specific neutralizing antibodies. While these immunized AG129 mice developed disease signs, they eventually recover from the immunization, compared to mock-immunized mice. Importantly, CHIKV-NoLS CAF01-immunized AG129 mice survive from subsequent CHIKV challenge and do not develop CHIKV-induced footpad swelling or disease. On the other hand, in immunocompetent C57/BL6 mice, CHIKV-NoLS CAF01-immunized mice developed delayed viremia at a similar titer compared to CHIKV-WT, and were protected from footpad swelling. However, immunization with either CHIKV-NoLS CAF01 or CHIKV-NoLS RNA produced significantly lower levels of neutralizing antibody compared to CHIKV-WT inoculation [126]. However, this study showed that the RNA-launched self-assembling viral particles generated immunity and protection that were just as strong as those of wild-type viral particles, suggesting the significant potential of this approach.

While multiple novel approaches have been explored to develop vaccines against alphaviruses, a potential prophylactic strategy that could be the development of a multivalent alphavirus vaccine given the reports of cross-neutralizing antibodies against conserved epitopes in the E2 protein across closely related alphaviruses. This approach would prove useful in endemic areas where alphavirus co-circulation occurs.

## Figures and Tables

**Table 1:** List of vaccine candidates against relevant alphaviruses currently under development ^1^.

Vaccine Against Virus	Name	Strain Vaccine Modelled After	Phase	Immunization			Challenge		Humoral Immune Response(s)	Ref
				Dose	Route	Schedule	Dose (Strain, Genotype)	Route		
Live-attenuated										
CHIKV, ONNV	RH-CHIKV EV-CHIKV RHEV-CHIKV	LR2006 OPY1	C57BL/6 mice, 3 week old	10^6^ PFU	s.c. in the ventral side of the right hind footpad	Single dose	10^6^ PFU LR2006 OPY1 or WT-ONNV IMTSSA/5163, 3 mpim	s.c. in the ventral side of the right hind footpad	IC50, 613 (RH-CHIKV), 3407 (EV-CHIKV), 921 (RHEV-CHIKV)	[130]
CHIKV	Δ5nsP3 (VLA1553-301 in clinical trials) and Δ6K	LR2006 OPY1	C57BL/6 mice, 5 to 6 week old	10^4^ or 10^5^ PFU	s.c. in both flanks	Single dose	10^6^ PFU LR2006 OPY1, 7 wpim	s.c.	NT50, 100 to 1000	[131,132,133]
			Cynomolgus macaques, 3–4 years old	10^5^ PFU	s.c. in the right upper back side	Single dose	100 AID50 (corresponding to 7000–10,000 PFU) LR2006 OPY1, 123 dpim	i.v.	NT50, >1000	
			Human clinical trial, Phase 1	3.2 × 10^3^, 3.2 × 10^4^ or 3.2 × 10^5^ TCID50	i.m.	Two doses (0 and 6 months, or 0 and 12 months)	NA	NA	GMT, 592.6 to 686.9	
CHIKV	CHIKV-NoLS	LR2006 OPY1	C57BL/6 mice, 21 days of age	10^4^ PFU	s.c.	Single dose	10^4^ PFU of LR2006 OPY1 or Ross River virus, 30 dpim	s.c.	<10% cells infected at 10^-1^ serum dilution	[127]
CHIKV	Stop CHIKV SuperStop CHIKV	LR2006 OPY1	C57BL/6 mice, 5 week old	10^4^ PFU	s.c.	Single dose	ND	ND	~5–25 (Stop CHIKV) and ~10–25 (SuperStop CHIKV) fold reduction compared to mock	[134]
CHIKV	ChikV HR	37997	C57BL/6 mice, 28 days of age	∼10^3^ PFU	s.c. into the left footpad	Single dose	10^3^ PFU CHIKV SL15649, 28 dpim	s.c. in the footpad	PRNT50, 5 to ~500	[135]
CHIKV	Heparin sulfate cell culture adapted	LR2006 OPY1	CD-1 mice, 21 days old	10^5^ GE	s.c. in the rear footpad	Single dose	10^3^ PFU LR2006 OPY1, 21 dpim	NA	~40 to 1000 fold change compared to mock	[136]
VEEV	V3526	IA/B Trinidad donkey	BALB/c, 6 to 8 week oldC3H/HeN mice, 6 to 8 week old	10^5^ PFU	s.c.	Single dose	10^5^ PFU of TrD, 28 dpim	NP	ND	[137,138,139,140]
			Cynomolgus macaques (age not specified)	2.5 × 10^6^ PFU	s.c.	Single dose	∼10^8^ PFU VEEV IE 68U201, 8 wpim	aerosol	PRNT80, 28 to 2560	
			Rhesus macaques (2 to 4 years old)	1.3 × 10^5^ or 7.5 × 10^4^ PFU	s.c. or i.t./i.s.	Single dose	ND	ND	PRNT80, ~80 to 300	
			Human clinical trial, Phase 1	25 or 125 PFU	s.c.	Single dose	NA	NA	NA	
VEEV	V4020	IA/B Trinidad donkey	BALB/c mice, 4 to 8 week old	10^4^ PFU	s.c.	Single dose	10^4^ PFU of VEEV TrD, 28 dpim	s.c.	PRNT80, 160 to1280	[141,142]
			Cynomolgus macaques (age not specified)	~10^4^ PFU	s.c. in the right leg	Single dose (or second dose at 2 x 10^4^ PFU i.m. if did not seroconvert)	10^6^ to 10^7^ PFU of the VEEV TrD, 73 dpim	aerosol	PRNT80, >640	
EEEV	5′U4&6 C65-69 E71-77 3′U11337 mutants	FL93-939	CD-1 mice, 5 to 6 week old	1.5 × 10^5^ GE	s.c. in footpad, or i.c.	Single dose	10^5^ PFU EEEV FL93, 21 dpim	s.c. in both footpads	PRNT80, 16 to ~4000	[143]
Live-attenuated (IRES)										
CHIKV	CHIKV/IRES	LR2006 OPY1	A129 mice, 3 or 10 week old	10^4^ PFU	i.d.	Single dose	100 PFU LR2006 OPY1, 94 dpim	i.d.	PRNT80, >320	[144,145]
			C57BL/6 mice, 3 week old	10^5^ PFU	s.c. in the hind leg	Single dose	10^6.5^ PFU Ross CHIKV, 21 dpim	i.n.	Mean PRNT80, 62	
			A129 mice, 8 to 10 week old	10^5^ TCID50	s.c.	Single dose	100 PFU LR2006 OPY1, 50 dpim	i.d.	Mean PRNT80, 1152	
			Cynomolgus macaques, >3 years old	10^5^ PFU	s.c. or i.d.	Single dose	10^5^ PFU LR2006 OPY1, 52 dpim	s.c. in the upper deltoid	PRNT80, 40 to 640PRNT50, 160 to 1280	
ONNV	CHIKV/IRES	LR2006 OPY1	A129 mice, 6 to 7 week old	10^4^ PFU	i.d.	Single dose	10^5^ PFU ONNV SG650, 38 dpim	i.d.	PRNT80, 160	[146]
VEEV	ZPC/IRESv1, ZPC/IRESv2	ID ZPC738	CD-1 mice, 6 to 8 week old	10^5^ PFU	s.c. in the scruff of the back	Single dose	10^5^ PFU VEEV 3908, 4 wpim	s.c. or aerosol	PRNT80, 40 to 324	[114]
			Cynomologous macaques, age not specified	10^5^ PFU	s.c. in the upper deltoid	Single dose	~ 8 × 10^5^ to 9 × 10^6^ PFU VEEV 3908, 35 dpim	aerosol	PRNT80, <20 to 20PRNT50, <20 to 160	
EEEV	EEE/IRES	FL93-939	NIH Swiss mice, 3 to 4 week old	10^4^ PFU	s.c. in the medial thigh	Single dose	10^3^ PFU of FL93-939, 4 wpim	i.p.	PRNT80, 160 to 640	[147]
VEEV	68U201/IRESv1 68U201/IRESv2	IE 68U201	CD1 mice, 6 to 8 week old	10^5^ PFU	s.c. in right hind leg	Single dose	(Lethal dose, NP) 68U201 at 1, 3, or 12 mpim	s.c.	PRNT80, 64 to ~300	[148,149]
			Cynomolgus macaques (age not specified)	10^5^ PFU	s.c. in the upper deltoid	Single dose	4 × 10^4^ PFU VEEV IE 68U201, 49 dpim	aerosol	PRNT80, ~100 to 340	
VEEV	VEEV/IRES/C	IA/B Trinidad donkey	CD-1 mice, 8 week old	10^5^ PFU	s.c.	Single dose	10^4^ PFU of VEEV 3908, 6 wpim	s.c.	Mean PRNT80, 184	[150]
MAYV	MAYV/IRES	MAYV-CH	BALB/c, 6 week old	2 × 10^5^ PFU	s.c. i.pl. route	Single dose	2 × 10^5^ PFU of WT MAYV, 28 dpim	s.c. i.pl. route	PRNT50, >640 (at 21dpi)	[146,151,152]
			AG129	2 × 10^4^, 2 × 10^3^ or 2 × 10^2^ PFU	s.c. i.pl. route	Single dose	2 × 10^3^ PFU of WT MAYV, 14 dpim	s.c. i.pl. route	ND	
			CD-1, 28-day old	10^5^ PFU	s.c. over the dorsum	Single dose	ND	ND	PRNT80, 160 to ≥ 640	
			AG129, 5 to 8 week old	10^4^ PFU	i.d. on the left foot	Single dose	10^4^ PFU of WT MAYV, 29 dpim	s.c.	PRNT80, 320 to ≥ 640	
Inactivated										
CHIKV	Vero cell adapted	DRDE-06	Swiss albino mice, 3 to 4 week old	10, 25 or 50 ug	s.c.	Three doses (0, 14 and 28 days)	ND	ND	PRNT90, 6400	[153]
CHIKV	BPL/formalin-inactivated CHIKV BBV87 (in clinical trials)	IND-06-AP3	BALB/c mice, 4 to 6 week old	10, 20 or 50 μg	i.m.	Two doses (0 and 14 days)	2.5 x 10^4^ TCID50 IND-06-AP3, 4 or 22 wpim	i.n.	GMT, NT50, 80 to 1280	[154]
			Human clinical trial, Phase 1	10, 20 or 30 μg	i.m.	Three doses (0, 29 and 57 days)	NA	NA	NA	[155]
RRV	Vero cell culture-derived whole-virus RRV vaccine Ross River Virus (RRV) Vaccine	T48	CD-1 mice, 7 to 8 week old	0.0025, 0.01, 0.039, 0.156, 0.625, 2.5 or 10 μg	s.c.	Two doses (0 and 28 days)	10^6^ TCID50 RRV T48, 42 dpim	i.v.	Mean NT, ≤2.9 to 46.2	[73,115,128,129]
			A129 mice, 7 to 8 week old	0.063, 0.25 or 1 μg	i.m.	Two doses (0 and 21 days)	10^2.5^ TCID50 T48, 42 dpim	s.c. into left footpad	Mean NT, ≤14 to 21	
			CD-1 mice, age not specified	10 μg	s.c.	Two doses (0 and 28 days)	10^6^ TCID50 T48, 6 wpim	i.v.	1000 TCID50	
			Guinea pigs (Duncan Hartley), age not specified	10 μg	s.c.	Single or two doses (0 and 6 weeks)	10^6^ TCID50 T48, 10 or 34 wpim	i.v.	NP	
			Human clinical trial, Phase 1/2	1.25, 2.5, 5, or 10 μg	i.m.	Three doses in escalation (0, 21 days, 6 months)	NA	NA	GMT, 50 to 520.9	
			Human clinical trial, Phase 3	2.5 ug	i.m.	Three doses (0, 3 weeks, 6 months)	NA	NA	μNT GMT, ~0 to 85	
EEEV	TSI-GSD-104 (formalin inactivated)	PE-6	Human clinical trial, Phase 2	NP	s.c. (0 and 28 days), i.d. (6 months)	Three doses (0, 28 days and 6 months)	NA	NA	PRNT80 >40 in 60% subjects (primary doses) versus 84% subjects (completed the 2-dose primary series and the 6-month dose)	[156,157,158]
EEEV	fCVEV1219 iCVEV1219 gCVEV1219	CVEV1219	BALB/c mice, 6 to 8 week old	0.1 to 5 µg of inactivated EEEV	i.n., s.c. or i.m.	Single dose or two doses (0 and 28 days)	Lethal dose of EEEV FL93-939, at 28 dpim (single dose) or 56 dpim (two doses)	aerosol	PRNT80, ~1 to 1000	[159]
VEEV	V3526 virus	V3526	BALB/c mice, 6 week old	0.2 μg (s.c.) or 0.04 μg (i.m.)	s.c. or i.m.	Two doses (0 and 28 days)	10^4^ PFU VEEV TrD, 56 dpim	aerosol or s.c.	GMT PRNT80, ~60 to 2500	[160]
VEEV	F-iV3526	V3526	BALB/c mice, 8 to 10 weeks old	1, 3 or 5 μg	i.n., s.c. (under the skin over the neck) or i.m. (thigh muscle of the hind leg)	Single dose	454 (i.n.), 897 (i.m.) or 55 (s.c.) PFU VEEV-TrD, 56 dpim	aerosol	Microneutralization titer of 100 to 3500	[161,162]
Virus-like particle										
CHIKV	VRC 311 Or VRC-CHKVLP059-00-VP/ PXVX0317 (in clinical trials)	37997	BALB/c mice, 6 to 8 week old	19 μg	i.m.	2 doses (2 and 5 weeks)	ND	ND	IC50, 10703 to 54600	[116,117,118,163]
			Cynomolgus macaques, 3 to 4 years old	20 μg	i.m.	3 doses (0, 4 and 24 weeks)	10^10^ PFU LR2006 OPY1, 15 wpim	i.v.	IC50, 10219 to 15072	
			Human clinical trial, Phase 1	10, 20 or 40 μg	i.m.	3 doses (0, 4 and 24 weeks)	NA	NA	IC50, 4525 to 8745	
			Human, clinical trial Phase 2	20 μg	i.m.	2 doses (0 and 28 days)	NA	NA	EC50 GMT, 2005	
			Human clinical trial (Phase 2b, recruitment completed)	6, 10 or 20 μg	NP	Two doses (0 and 14 or 28 days)	NA	NA	NA	
CHIKV	Baculovirus-expressed VLP	S27	AG129, 6 week old	1 μg	s.c.	2 doses (0 and 21 days)	1000 TCID50 S27, 6 wpim	i.p.	PRNT95, 40 to 80	[164,165]
			C57BL/6 mice, 6 to 12 week old	0.1 or 1 μg	s.c.	Single dose	10^4^ CCID_50_ LR2006 OPY1, 6 wpim	s.c.	NT95, ~1,100	
CHIKV	Yeast-expressed VLP	DRDE06/DRDE07	BALB/c mice, 4 week or 2 days old	10, 20 or 40 ug	s.c.	Three doses (0, 14 and 28 days)	ND	ND	NT50, 128 to 2048	[166]
VEEV	Venezuelan Equine Encephalitis Monovalent Virus-Like Particle Vaccine (VEEV)	NA	Human clinical trial (Phase 1, not recruiting)	2, 10, or 20 μg	i.m.	Dose escalation (0, 28 days, and day 140 booster)	NA	NA	NA	[167]
WEEV, EEEV, and VEEV	VRC-WEVVLP073-00-VP (Trivalent vaccine)	WEEV CBA87, EEEV PE-6 and VEEV TC-83	BALB/c mice, 6 to 8 week old	monovalent (5 μg) or trivalent (5 μg each)	i.m.	Two doses (0 and 21 days)	2.5 × 10^3^ PFU WEEV CBA87, 8.9 × 10^3^ PFU EEEV FL93-939, and 1.3 × 10^3^ PFU VEEV Trinidad donkey, 56 dpim	aerosol	PRNT80, ~250 to 100000	[168]
			Cynomolgus macaques, age not specified	Monovalent (20 μg) or trivalent (20 μg each)	i.m.	Two doses (0 and 28 days)	10^6^ PFU WEEV CBA87, 10^8^ PFU EEEV FL93-939, and 10^8^ VEEV Trinidad donkey, 56 dpim	aerosol	PRNT80, ~1000 to 10000	
			Human clinical trial, Phase 1	6, 30 or 60 μg	i.m.	Dose escalation (0 and 8 weeks)	NA	NA	NA	[169]
DNA/RNA										
VEEV	VEEV 26S DNA plasmid	I/AB TrD	BALB/c mice, 6 to 8 week old	∼3 μg	DNA/gene gun, delivered to two sites on the abdomen of each mouse	Three doses (at 3-week intervals)	∼10^4^ PFU of TrD, 9 wpim	s.c., aerosol	PRNT50, GMT <1.6 to 2.5	[170,171]
			Hartley guinea pigs, age not specified	~5 μg	DNA/gene gun, delivered to two sites on the abdomen of each mouse	Three doses (0, 4 and 8 weeks)	∼10^4^ PFU of TrD, 21 wpim	aerosol	PRNT50, 0 to 640	
VEEV	DNA-Ad	TC-83	BALB/c mice, 6 to 8 week old	1 μg of DNA per dose and 107 PFU of RAd/VEEV #3 per boost	gene guni.n.	immunised with the DNA vaccines on day 0, 14 and 28 and Ad-based vaccine on day 42	100 LD50 of virulent airborne VEEV, 63 dpim	aerosol	PRNT50, 160	[172]
VEEV	AG4-1C7 AG4-1G2 AG2-5A7 AG2-5A10 plasmid DNA	I/AB TrD	BALB/c mice, 6 to 8 week old	4 μg	particle-mediated epidermal delivery (i.d.)	Three doses (at 3-week intervals)	∼10^4^ PFU of VEEV TrD (≥1000 LD50), 70 dpim	aerosol	PRNT80, ~1 to 5.5 log_10_ GMT	[173]
VEEV	pTC83 iDNA	TC-83	BALB/c mice, 3 week old	50 μg	i.m. electroporation	Single dose	10^5^ PFU VEEV 3908, 21 dpim	s.c.	PRNT80, 10 to 320	[174]
WEEV	pE3-E2-6K-E1 pE3-E2 P6K-E1	71V-1658	BALB/c, age not specified	2 μg	gene gun	Three doses (14 days apart)	1500 PFU WEEV 71V-1658, Fleming, or CBA87, 42 dpim	i.n.	ND	[175]
CHIKV	pCHIKV-Capsid, pCHIKV-Envelope (pMCE321)	Consensus	C57BL/6 mice, 3 to 4 week old	25 µg, 2–3 times	Electroporation	Two doses (2 weeks apart)	ND	ND	ND	[176,177,178]
			C57BL/6 mice, 6 to 8 week old	25 μg	i.m. electroporation	Three doses (0, 14 and 21 days)	7log_10_ PFU of PC-08, 35 dpim	i.n.	NP	
			BALB/c mice	25 μg	i.m. electroporation	Two doses (2 weeks apart)	7log_10_ PFU PC-08	i.n.	TCID50, 20 to 320	
			Rhesus macaques, age not specified	1 mg	i.m. electroporation	Three doses (4 weeks apart)	ND	ND	TCID50, 80 to 1280	
CHIKV	Δ5nsP3 and Δ6K DNA	LR2006 OPY1	C57BL/6 mice, 5 to 6 week old	20 μg	i.d. with DermaVax electroporation	Single dose or two doses (0 and 3 weeks)	10^6^ PFU LR2006 OPY1, 7 wpim	s.c.	NT50, 100 to 10000	[131]
CHIKV	CHIKV-NoLS RNA	LR2006 OPY1	C57BL/6 mice, 28 days of age	2 μg	s.c. in the ventral/lateral side of the right foot	Single dose	10^4^ PFU LR2006 OPY1, 30 dpim	s.c. in the ventral/lateral side of the right (ipsilateral) or left (contralateral)	PRNT80, 0	[126]
			AG129 mice, 28 days old	2 μg	s.c. in the ventral/lateral side of the right foot	Single dose	10^4^ PFU LR2006 OPY1, 30 dpim	s.c. in the ventral/lateral side of the right (ipsilateral) or left (contralateral)	ND	
VEEV, WEEV and EEEV	3-EEV	VEEV IAB TrD, WEEV CBA874 and EEEV FL91-46794	C57BL/6 mice, 6 to 8 week old	15 μg	i.m. electroporation	Two doses (0 and 21 days)	10^4^ PFU VEEV IAB TrD or 2 × 10^4^ PFU WEEV CBA874 or 105 PFU EEEV FL91-46794, 7 wpim	aerosol	PRNT80, ~1 to 1000	[179]
MAYV	scMAYV-E	NA	C57BL/6 mice, 5 to 8 week old	25 μg	i.m. electroporation	Single, two doses or three doses (at 2 week intervals)	ND	ND	PRNT50, 789.8	[180]
			A129 mice, 4 to 6 week old	25 μg	i.m. electroporation	Single, two doses or three doses (at 2 week intervals)	10^2^ PFU MAYV 15537	i.p.	ND	
CHIKV	p181/25-7	TSI-GSD-28	BALB/c mice, 3 week old	10 μg	i.m. electroporation	Single dose	6 × 10^6^ PFU CHIKV Ross, 28 dpim	i.n.	PRNT80, 160 to 1280	[181]
CHIKV	dMaB	NA	BALB/c mice, age not specified	100 μg	Electroporation	Single dose	10^7^ PFU Del-03	s.c. or i.n.	IC50, 3 to 4.5log_10_	[182]
CHIKV	iRNAΔ5nsP3 iDNAΔ5nsP3	LR2006 OPY1	C57BL/6 mice, 8 week old	0.125, 1.25 or 10 μg	i.m. in the gastrocnemius muscle of the left hind leg	Single dose	10^6^ PFU LR2006 OPY1, 5 wpim	s.c. at the dorsal side of each hind foot	NT50, ~1 to 10^4^	[183]
VEEV	pMG4020 DNA plasmid	TC-83	BALB/c, 4 to 8 week old	0.5 or 5 ug	i.m. electroporation	Single dose	10^4^ PFU VEEV TrD, 28 dpim	s.c.	PRNT80, 320 to >1280	[141]
VEEV	VEEV_WT_ VEEV_COCAP_ VEEV_CO_	IAB TrD	BALB/c, 6 to 8 week old	25, 5, or 1 μg	i.m. electroporation	Two doses (3 weeks apart)	∼10^4^ PFU VEEV IAB strain TrD, 7 wpim	aerosol	PRNT80, 1 to ~4.5log_10_	[184,185]
			New Zealand White rabbits, age not specified	500 μg of VEEV_CO_	i.m. electroporation	Three doses (0, 28 and 230 days)	ND	ND	PRNT80, ~3log_10_ to 5log_10_	
			Cynomolgus macaques, age not specified	50 or 500 μg of VEEV_CO_	i.m. electroporation	Two doses (0 and 56 days)	3 × 10^8^ PFU VEEV IAB TrD	aerosol	PRNT80, ~0.8log_10_ to 3.5log10	
			Human clinical trial, Phase 1	0.5 or 2 mg	i.m. electroporation or i.d. electroporation	Three doses (days 0, 28, and 56)	NA	NA	GMT PRNT80, 7 to 78	
WEEV	pVHX-671V-1658 pVHX-6 CBA87 pVHX-6 Fleming	Fleming, CBA 87 or 71V-1658,	BALB/c mice, age not specified	2 shots × 2.5 μg precipitated on 0.5 mg gold	gene gun	Four doses (2 weeks apart)	1.5 × 10^3^ PFU WEEV Fleming, CBA 87 or 71V-1658, 8 wpim	i.n.	ND	[186]
WEEV and EEEV	LANAC E1ecto	WEEV McMillan	CD-1 mice, 4 to 6 week old	10 μg	s.c. injection dorsal to the cervical spine	Two doses (2 weeks apart)	10^4^ PFU WEEV McMillan, Montana-64, or EEEV Florida-93, 4, 5, 9, 11, or 13 wpim	i.n. or s.c.	PRNT50, <40 to 200	[187]
CHIKV	mRNA-1388 (or VAL-181388 in clinical trials)	NA	Human clinical trial, Phase 1	25, 50 or 100 μg	i.m.	Dose escalation procedure (0 and 4 weeks)	ND	ND	‘dose-dependent increase’ in neutralizing and binding antibody titers	[188]
CHIKV	mRNA-1944	SL15649	AG129, age not specified	0.4, 1 or 10 mg/kg	i.v. tail vein injection	Single dose	10^2.5^ TCID50 of CHK	subcutaneous injection in the footpad and hock of the right leg	ND	[189,190]
			Cynomolgus macaques, 2 to 3 year old	0.5 mg/kg	i.v.	Single dose	ND	ND	FRNT50, 5 to 12	
			Human clinical trial, Phase 1 (active, not recruiting)	0.1, 0.3 and 0.6 mg/kg	i.v.	Dose escalation	NA	NA	NT50, ‘all participants also showed circulating neutralizing antibody activity’	
Subunit										
CHIKV	CHIKV-sE1 and -sE2	S27	AG129 mice, 6 week old	2 μg	s.c.	Two doses (0 and 21 days)	1000 TCID50 of S27 isolate, 9 wpim	i.p.	NT95, <25	[164,165,191]
CHIKV	rE2p	IND-06-AP3	BALB/c, 6 to 8 week old	10, 20 or 50 μg	i.m.	Two doses (2 weeks apart)	Mice immunized with 50 μg challenged with 7 log10 TCID50 /mL, 4 or 22 wpim	i.n.	NT50, 0.25log10 to 2.5log10	[154]
CHIKV	CHIKE1 and CHIKE2 recombinant proteins	DRDE-06	BALB/c	40 μg	s.c.	Three doses (0, 21 and 35 days)	ND	ND	PRNT90, 32 to 512	[192]
Chimeric virus										
Measles virus-based chimeras										
CHIKV (VLP)	MV-CHIKV	06–49	CD46-IFNAR, 6 week old	10^3^ to 10^5^ TCID50	i.p.	Single or two doses (30 days apart)	100 PFU of CHIKV 06-49, 2 mpim	i.p.	PRNT50, 450 to 4050 PRNT90, 50 to 450	[193,194,195,196,197]
			Cynomolgus macaques, age not specified	5 × 10^5^ (± 0.5 log) TCID50	i.m.	Two doses (28 days apart)	1.4 × 10^5^ PFU LR2006 OPY1, 56 dpim	s.c.	PRNT80, 40 to >640	
			Human clinical trial, Phase 1	1.5 × 10^4^, 7.5 × 10^4^ or 3.0 × 10^5^ TCID50	i.m. or s.c.	Dose escalation (0 and 28 days, or 0 and 90 days)	NA	NA	PRNT50, 5 to 433	
			Human clinical trial, Phase 2	5 × 10^4^ or 5 × 10^5^ TCID50	i.m.	Three doses (0, 28, and 196 days)	NA	NA	PRNT50, ~5 to 5000	
Alphavirus-based chimeras										
CHIKV	VEE/CHIKV EEE/CHIKV SIN/CHIKV	LR2006 OPY1	NIH Swiss, C57BL/6, >3 week old	5.8 log_10_ PFU (VEE/CHIKV and SIN/CHIKV), 5.3 log_10_ PFU (EEE/CHIKV)	s.c. in the medial thigh	Single dose	6.5 log_10_ PFU (Ross CHIKV strain), 21 dpim	i.n.	PRNT80, 20 to 320	[198]
CHIKV	VEE/IRES-CHIKV VEE/IRES-C/CHIKV	NA	A129 mice, 6 to 9 week old	10^4^ PFU	s.c.	Single dose	10^2^ PFU of LR2006 OPY1, 5 weeks post immunization	s.c.	PRNT80, >640	[199]
CHIKV	EILV-CHIKV	CHIKV 996659	C57BL/6 mice, 4 week old	8.8 log_10_ PFU	s.c.	Single dose	6 log_10_ PFU 99659, 30 dpim	i.d.	PRNT80, ≥ 80	[125,220]
			IFNα/βR−/−, 6 week old	8.8 log_10_ PFU	s.c.	Single dose	3 log_10_ PFU 99659, 292 dpim	i.d.	PRNT80, 160 to 1280	
			Cynomolgus macaques, 3 to 5 years	8.1 log_10_ PFU	i.m. into the right quadriceps	Single dose	5 log_10_ PFU LR2006 OPY1, 31 dpim	s.c.	PRNT80, 80 to 640	
EEEV	EILV/EEEV	EEEV FL-93	Adult CD-1 mice (age not specified)	10^8^ PFU	s.c.	Single dose	10^5^ PFU EEEV-FL93, 70 dpim	i.p.	PRNT80, 80 to 640	[125,220]
EEEV	Trivalent EILV/EEEV EILV/VEEV EILV/CHIKV	EEEV FL-93, VEEV IAB TC-83, CHIKV 996659	Adult CD-1 mice (age not specified)	10^8^ PFU	s.c.	Single dose	10^5^ PFU EEEV-FL93, 70 dpim	i.p.	PRNT80, 40 to 640 and 20 to 640 for mono- and trivalent vaccines respectively	
VEEV	EILV/EEEV	VEEV IAB TC-83	Adult CD-1 mice (age not specified)	10^8^ PFU	s.c.	Single dose	10^3^ PFU VEEV-IC 3908, 70 dpim	s.c.	PRNT80, 80 to 1280	
VEEV	Trivalent EILV/EEEV, EILV/VEEV EILV/CHIKV	EEEV FL-93, VEEV IAB TC-83, CHIKV 996659	Adult CD-1 mice (age not specified)	10^8^ PFU	s.c.	Single dose	10^3^ PFU VEEV-IC 3908, 70 dpim	s.c.	PRNT80, 40 to 640 and 20 to 80 for mono- and trivalent vaccines respectively	
EEEV (Sindbis virus)	SIN/NAEEEV	EEEV FL93-939	NIH Swiss mice, 8 week old	3.7, 4.7 or 5.7 log_10_ PFU	s.c.	Single dose	6 log_10_ PFU FL93-939, 28 dpim	i.p.	PRNT80, 125 to 660	[200]
	SIN/SAEEEV	EEEV BeAr436087	NIH Swiss mice, 8 week old	3.8, 4.8 or 5.8 log_10_ PFU	s.c.	Single dose	6 log_10_ PFU FL93-939, 28 dpim	i.p.	PRNT80, 28 to 308	
VEEV	SIN-83	VEEV IAB TC-83	Weanling NIH Swiss mice, 6 day old	10^3^, 10^4^, 10^5^ or 10^6^ PFU	s.c.	Single dose	10^6^ PFU VEEV IC ZPC738 IC SH3	s.c.in medial thigh	PRNT80, 30 to 960	[201,202]
			NIH Swiss mice, 6 week old	5 × 10^5^ PFU	s.c.	Two doses	2 x 10^5^ or 10^6^ PFU VEEV ZPC738, 8 wpim	s.c., i.c., or i.n.	PRNT80, 55 to 73 (single), 100 to 160 (booster)	
	SAAR/TRD	VEEV IAB TrD	NIH Swiss mice, 6 week old	5 × 10^5^ PFU	s.c.	Two doses	2 x 10^5^ or 10^6^ PFU VEEV ZPC738, 8 wpim	s.c., i.c., or i.n.	PRNT80, 126 to 167 (single), 152 to 160 (booster)	
	SIN/TRD	VEEV IAB TrD	NIH Swiss mice, 6 week old	5 × 10^5^ PFU	s.c.	Two doses	2 x 10^5^ or 10^6^ PFU VEEV ZPC738, 8 wpim	s.c., i.c., or i.n.	PRNT80, 37 to 57 (single), 50 to 73 (booster)	
	SIN/ZPC	VEEV ID ZPC738	NIH Swiss mice, 6 week old	5 × 10^5^ PFU	s.c.	Two doses	2 x 10^5^ or 10^6^ PFU VEEV ZPC738, 8 wpim	s.c., i.c., or i.n.	PRNT80, 187 to 253 (single), 253 to 487 (booster)	
	All the above	VEEV IAB TC-83, IAB TrD, ID ZPC738	Syrian golden hamsters, 6 week old	5 × 10^5^ PFU	s.c. in the medial thigh	Single dose	10^6^ PFU	s.c.in medial thigh	ND	
WEEV	SIN/CO92	WEEV CO92-1356	NIH Swiss mice, 6 week old	3.5, 4.5, or 5.0 log_10_ PFU	s.c. in the medial thigh	Single dose	5.3 log_10_ PFU WEEV TBT235, 28 dpim	i.n.	PRNT80, 20 to 640	[203]
	SIN/SIN/McM	WEEV McMillan	NIH Swiss mice, 6 week old	4.8 or 5.8 log_10_ PFU	s.c. in the medial thigh	Single dose	5.0 log_10_ PFU WEEV McMillan, 28 dpim	i.n.	PRNT80, 600 to 604	
	SIN/EEE/McM	EEEV 436087 and WEEV McMillan	NIH Swiss mice, 6 week old	4.6 or 5.6 log_10_ PFU	s.c. in the medial thigh	Single dose	5.0 log_10_ PFU WEEV McMillan, 28 dpim	i.n.	PRNT80, 416 to 420	
Vaccinia virus-based chimeras										
CHIKV	MVA-CHIKV	LR2006-OPY1	C57BL/6 mice, 6 to 8 week old	10^7^ PFU (first dose), 2 × 10^7^ PFU (second dose)	i.p.	Two doses (2 weeks apart)	10^6^ PFU LR2006-OPY1, 9 wpim	s.c. in the dorsal side of each hind foot	NT50, ~100 to 3000	[204]
CHIKV	MVA-CHIK	LR2006-OPY1	BALB/c mice, 4 to 6 week old	10^7^ TCID50 units	i.d. injection into the left hind footpad.	Single or two doses (28 days apart)	10^4^ LR2006 OPY1 TCID50 units at 39 or 42 dpim	i.d.	TCID50, 5 to 15	[205]
			AG129, 6 to 10 week old	10^7^ TCID50 units	i.d. injection into the left hind footpad.	Single or two doses (28 days apart)	10^2^ LR2006 OPY1 TCID50 units at 39 or 42 dpim	i.d.	TCID50, 4 to 8	
CHIKV	MVA-6KE1, MVA-E3E2, MVA-6KE1E3E2	CHIKV S27	AG129 mice, 7 week old	5 × 10^6^ TCID50	i.m. into the quadriceps muscles of the left leg	Two doses (3 weeks apart)	10^3^ TCID50 CHIKV-S27 and CHIKV-IND/NL10, 63 dpim	i.p.	NT100, 10 to 160	[206]
EEEV, VEEV, and WEEV	MVA-BN-E/V/W (monovalent) MVA-BN-E + MVA-BN-V + MVA-BN-W (triple mixture of monovalent vaccines) MVA-BN-WEV (trivalent)	WEEV 71 V-1658, EEEV FL93-939NA and VEEV TrD	BALB/c mice, age not specified	10^8^ TCID50	s.c. or i.m.	Two doses (28 days apart)	5 × 10^3^ or 10^4^ PFU of WEEV Fleming, EEEV PE6, or VEEV TrD, 14 days post booster	i.n.	NT50, ~750 to 3800 (monovalent), ~<60 to 340 (triple mixture of monovalent vaccines) and ~<60 to 380 (trivalent)	[207]
Adenovirus-based chimeras										
CHIKV	CAdVax-CHIK	LR2006 OPY1	CD-1 or C57BL/6, 6 to 8 week old	10^8^ IU	i.p.	Single dose	10^4^ CCID50 LR2006 OPY1 or QIMR, 6.5 wpim	s.c. into side of each hind foot towards the ankle	NT100, ~2000	[208]
CHIKV	ChAdOx1 Chik	NA	BALB/c, 6 to 8 week old	10^8^ IU	i.m.	Single dose	ND	ND	NT50, 5.39 × 10^3^	[209,210]
			AG129, 5 week old	10^8^ IU	i.m. in each leg	Single dose	9.7 × 10^4^ PFU LR2006 OPY1, 30 dpim	i.d. into the left foot	ND	
	ChAdOx1 Chik ChAdOx1 Chik ΔCap		AG129, 5 week old	10^8^ IU	i.m. in each hind leg	Single dose	9.7 × 10^4^ PFU of LR2006 OPY1, 30 dpim	i.d. into the left foot towards the ankle	PRNT80, 32 to 64 (Chik), 16 to 32 (Chik ΔCap)	[211]
	CHIK001 (in clinical trials)		Human clinical trial, Phase 1	5 × 10^9^, 2.5 × 10^10^ or 5 × 10^10^ vp	i.m.	Single dose	ND	ND	ND	[212]
MAYV	ChAdOx1 May	NA	AG129, 5 week old	1.6 × 10^4^ PFU	i.m. in each leg	Single dose	1.6 × 10^4^ PFU MAYV-CH, 30 dpim	i.d. into the left foot	PRNT50, 160 to 620	[210]
VEEV	Rad/VEEV#3	VEEV IAB TC-83	BALB/c, 6 to 8 week old	10^7^ PFU	i.n.	Three doses (at 0, 7 and 21 days)	Dose ND, 28 dpim	aerosol	PRNT50 (NP)	[213]
			BALB/c, 6 to 8 week old	10^7^ PFU	i.n.	Two doses (at 0, 21 days)	5000 LD50 TrD, 42 dpim	aerosol	ND	[214]
WEEV	Ad5-WEEV	WEEV 71V-1658	BALB/c mice, age not specified	10^7^ PFU	i.m.	Single or two doses (at 4 weeks)	1.5 × 10^3^ PFU Fleming or 71V-1658, 13 wpim	i.n.	PRNT50, 160	[215]
WEEV	Ad5-E1	WEEV 71V-1658	BALB/c mice, 6 to 9 week old	10^7^ PFU	i.m. in both leg	Single dose	50 LD50 of 71V-1658, 7 dpim, or 400 LD50 CBA87, 1, 3, 5 or 7 dpim	i.n.	PRNT50, <10	[216]
Vesiculovirus-based chimeras										
CHIKV	rVSVΔG-CHIKV	CHIKV S27	C57BL/6, 3 week old	10^6^ PFU	i.m. into the right hind leg muscle	Single dose	10^4^ PFU LR 2006 OPY1, 30 dpim	s.c. in the left rear footpad	PRNT80, 80 to 640	[217]
VEEV	rVSIV-VEEV	VEEV ZPC738	CD-1, 4 to 6 week old	10^8^/10^7^ PFU	i.m.	Single dose	10^4^ PFU VEEV ZPC738, 35 or 245 dpim	s.c.	PRNT80, 288 to 600 at 25 and 35 dpim, 304 to 360 at 245 dpim	[218]
VEEV	rISFV-VEEV	VEEV ZPC738	CD-1, 4 to 6 week old	10^8^ PFU	i.m.	Single dose	10^4^ PFU VEEV ZPC738, 28 dpim	s.c.	PRNT80, ≥20	
			CD-1, 4 to 6 week old	10^8^ PFU	i.m.	Single dose	10^4^ PFU VEEV ZPC738, 35 or 245 dpim	s.c.	PRNT80, 40 to 160 at 25 and 35 dpim, 25 to 64 at 245 dpim	
EEEV	rISFV-EEEV	EEEV FL93-939	CD-1, 4 to 6 week old	10^8^ PFU	i.m.	Single dose	10^4^ PFU EEEV FL93-939, 28 dpim	s.c.	PRNT80, ≥20	
Epitope-based										
CHIKV	E2EP3	NA	C57BL/6 mice, 3 week old	100 μg (50 μg for booster doses)	s.c. in the abdominal flank	Three doses (0, 14 and 21 days)	10^6^ PFU CHIKV SGP11, 30 dpim	s.c. region at the ventral side of the right hind footpad, towards the ankle	~40% reduction from mock control	[23]

^1 ^s.c., subcutaneous; i.v., intravenous; i.m., intramuscular; i.d., intradermal; i.p., intraperitoneal; i.n., intranasal; i.t./i.s., intrathalamic/ intraspinal; i.pl., intraplantar; i.c., intracranial; dpim, days post immunization; wpim, weeks post immunization; mpim, months post immunization; IRES, internal ribosome entry site; PFU, plaque forming units; TCID50, 50% tissue culture infective dose; CCID50, 50% cell culture infectious dose; IC50, 50% inhibitory concentration; GE, genomic equivalents; IU, infectious units; AID50, 50% animal infectious dose; PRNT50, 50% plaque reduction neutralizing antibody titer; PRNT80, 80% plaque reduction neutralizing antibody titer; PRNT90, 90% plaque reduction neutralizing antibody titer; LD50, median lethal dose; NT50, 50% neutralizing titer; GMT, geometric mean titer; μNT, neutralizing titer; SIN, Sindbis virus; ISFV, Isfahan virus; May, Mayaro virus; EILV, Eilat virus, VSV/VSIV, vesicular stomatitis virus; MV, measles virus; MVA, modified vaccinia virus Ankara; NP, not provided; NA, not applicable; WT, wild type. Data curated from literature reported through February 2021.

## Data Availability

No new data were created or analyzed in this study. Data sharing is not applicable to this article.

## Supplementary Materials

The following are available online at https://www.mdpi.com/article/10.3390/microorganisms9050899/s1. Supplementary Figure S1. Alignment of E1 and E2 amino acid sequences of arthritogenic and encephalitic alphaviruses.
