# Supplementary material for: Insights into Antibody-Mediated Alphavirus Immunity and Vaccine Development Landscape
Source: Microorganisms. 2021 Apr 22;9(5):899. doi: 10.3390/microorganisms9050899 (PMC8145166; doi:10.3390/microorganisms9050899)
Supplement: Supplementary file 1 [file microorganisms-09-00899-s001.zip › microorganisms-1169685-supplementary.pdf]

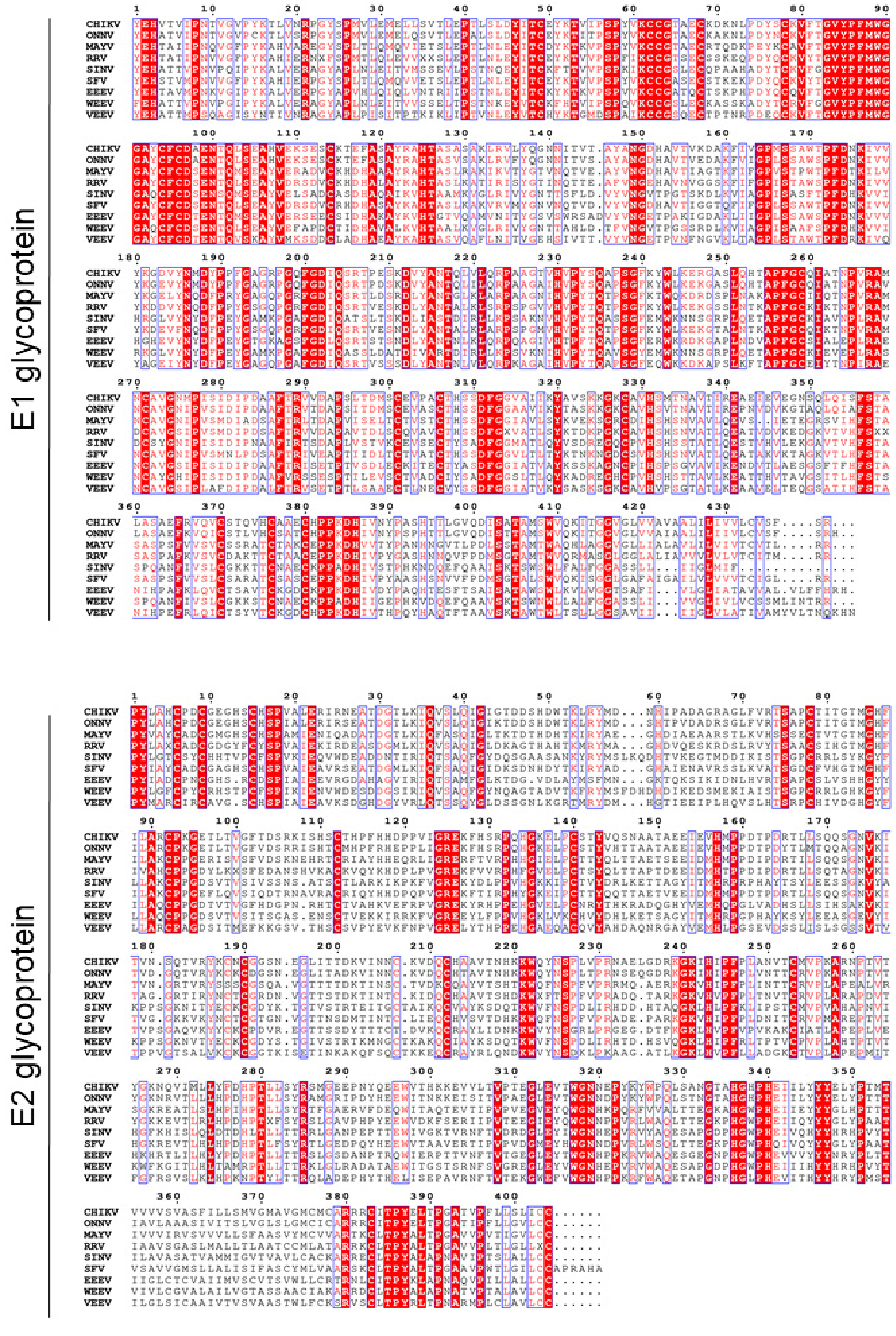

Figure S1. Alignment of E1 and E2 amino acid sequences of arthritogenic and encephalitic alphaviruses. Alignment of E1 and E2 glycoproteins of reference arthritogenic (CHIKV: NC_004162.2, ONNV: NC_001512.1, MAYV: NC_003417.1, RRV: NC_001544.1, SINV: NC_001547.1, SFV: NC_003215.1) and encephalitic (EEEV: NC_003899.1, WEEV: NC_003908.1, VEEV: NC_001449.1) alphaviruses. 100% conserved residues are highlighted in red (white letters), red letters indicate homologous residues. Blue boxes demarcate highly similar regions between sequences, black letters are non-conserved residues. Reference sequences were aligned in PROMALS3D and ESPript 3.0. was used to prepare the figure.
